# Validity of self-assessment pubertal Tanner stages by realistic color images and Pubertal Development Scale in a longitudinal cohort study

**DOI:** 10.3389/fped.2024.1380934

**Published:** 2024-07-16

**Authors:** Jie Luo, Di Wu, Yu Tian, Yujie Wang, Qin Zhang, Zongwei He, Hong Wang, Qin Liu

**Affiliations:** ^1^ School of Public Health, Research Center for Medicine and Social Development, Chongqing Medical University, Chongqing, China; ^2^ College of Medical Informatics, Chongqing Medical University, Chongqing, China

**Keywords:** self-assessment, Tanner stages, pubertal, reliability, validity

## Abstract

**Introduction:**

To date, the reliability of pubertal development self-assessment tools is questioned, and very few studies have explored the comparison between these tools in longitudinal studies. Hence, this study aimed to examine the reliability of pubertal development self-assessment using realistic color images (RCIs) and the Pubertal Development Scale (PDS) in a longitudinal cohort study.

**Methods:**

Our longitudinal study recruited 1,429 participants (695 boys and 734 girls), aged 5.8–12.2 years old, in Chongqing, China. We conducted two surveys, 6 months apart. Tanner stages were examined by trained medical students at each visit. RCIs and PDS scores were used to self-assess puberty at each visit. Agreement between physical examination and self-assessment was determined using weighted kappa (wk), accuracy, and Kendall rank correlation.

**Results:**

The concordance of puberty self-assessment using RCIs at baseline and the first follow-up was almost perfect in girls and boys, wk >0.800 (*p *< 0.001). At baseline, the concordance of genital development self-assessment using RCIs was fair in boys, wk = 0.285 (*p* < 0.001), and that of boys’ pubic hair development self-assessment using RCIs was poor, wk = 0.311 [95% confidence interval (CI) −0.157 to 0.818]. The wk of the PDS was less than 0.300, except for breast development. The reliability and validity of the PDS in this study population were low, and the consistency of the PDS was not good.

**Conclusions:**

The concordance of RCIs is better than that of the PDS. Pubertal development self-assessment using RCIs is reliable, while the reliability and validity of the PDS are unacceptable. Therefore, RCIs are recommended as a reliable pubertal development self-assessment tool to measure pubertal development for large-scale epidemiological investigations and long-term longitudinal studies in China.

## 1 Introduction

Puberty is a significant period of physiological changes and sexual maturity, marking the transition from childhood to adolescence. Extensive evidence indicates that abnormal development during this period is a contributing factor to obesity, hypertension, insulin resistance, type 2 diabetes, cardiovascular disease, cancers including breast cancer, endometrial cancer, and ovarian cancer, and emotional and behavioral problems (1–7). Therefore, monitoring pubertal development is significant for health during both childhood and adulthood.

Pubertal development requires accurate puberty measurements to determine temporal changes in pubertal milestone events. However, the measurement of pubertal development varies widely across studies, and there are many challenges in obtaining data for the assessment of pubertal development. The current gold standard for measuring puberty is the Tanner Sexual Maturation Scale (SMS) (8, 9). The SMS classifies development into five levels based on breast and pubic hair development for girls and genital and pubic hair development for boys: these levels include Tanner stage 1 (prepubertal), stage 2 (early pubertal), stage 3 (midpubertal), stage 4 (late pubertal), and stage 5 (postpubertal). The SMS requires the child to undress and be examined by a professional. Despite the accuracy provided by professionals, this method might be impractical in non-clinical settings, especially in large-scale epidemiological studies, due to its labor-intensive, material, financial, time-consuming nature, making it challenging to implement. In addition, because of the sensitivity of pubertal development assessment, children and parents may be less likely to agree to the SMS compared to the self-assessment of pubertal development. As a result, many studies rely on pictures or line drawings of Tanner stages that ask children or their parents to self-assess the children's pubertal development. The Pubertal Development Scale (PDS) (10) is another widely used tool for self-assessment of pubertal development, designed to avoid the sensitivity issues of visual sexual maturation charts.

Although pubertal development self-assessment using pictures and the PDS are widely used, their reliability has been questioned. On the one hand, the results of a systematic review and meta-analysis (11) of 22 studies showed moderate or substantial agreement between clinician assessment and self-assessment using pictures. However, a US study (12) showed that self-assessment of Tanner stages using pictures is not a reliable method. On the other hand, the consistency between self-assessment by the PDS and physical examination (PE) remains controversial in different studies. Although several studies (13–17) indicated that the PDS could be used in large-scale epidemiological investigations of pubertal development, they reported varying levels of agreement—moderate agreement, fair agreement, and even slight agreement—between clinician assessment and self-assessment using the PDS. Hence, the reliability of pubertal development self-assessment tools is controversial, and the scientific accuracy and authenticity of the study results will be affected, although a large number of studies used pictures, especially the PDS, to self-assess pubertal development.

Moreover, few studies have compared the agreement between pictures and the PDS, which are both popular tools for self-assessment of pubertal development. Compared to pictures, the PDS is widely used, and it has been cited more than 2,500 times. While the reliability of the PDS is more controversial (16–18), we believe that the PDS and pubertal development pictures have not been compared in a single study. If the concordance of pictures is better than that of the PDS, pictures could potentially replace the PDS as the preferred self-assessment tool of pubertal development, ensuring scientific and authentic results. Therefore, studies comparing pictures with the PDS should be carried out, as this can assist doctors, healthcare providers, epidemiologists, and other researchers in deciding a more appropriate pubertal development self-assessment tool.

At present, global trends indicate that adolescent puberty is generally occurring earlier, which is a problem that needs significant attention (19, 20). Given that puberty is a process requiring longitudinal tracking and involves psychological sensitivity of children during this period (21, 22), pubertal development examination by SMS may increase the psychological burden on adolescents. Furthermore, cross-sectional studies cannot cover the processes of pubertal development, such as pubertal trajectory and tempo, which are important for health services for children and adolescents. Assessing pubertal development every 6 months provides a more precise method for tracking pubertal changes and is widely conducted in studies such as the Danish National Birth Cohort (23). With little known about the longitudinal reliability of pubertal development self-assessment tools, more evidence is needed to longitudinally track the reliability of the self-assessment tools.

To date, the reliability of pubertal development self-assessment tools is questioned, and few previous studies have explored the comparison between pictures and the PDS in longitudinal studies. Hence, this study included the most widely used self-assessment tools to verify whether pictures with Tanner stages and the PDS can be used in cohorts through a longitudinal study in Chongqing, China.

## 2 Methods

### 2.1 Participants

The study participants were from an ongoing longitudinal cohort aimed at exploring adolescents’ pubertal development and its influencing factors and health implications. During the enrollment period since May 2014, 1,237 participants (542 girls and 695 boys) were enrolled from grades 1–4 in primary schools in an urban district of Chongqing, China. After 6 months, we added a further 192 girls at the first follow-up and thus officially recruited 1,429 participants (734 girls and 695 boys) for this study, aged 5.8–12.2 years old; informed consent was obtained from their parents and themselves. JLP District in Chongqing is a pilot district for urban and rural planning, and its demographic characteristics and economic status are similar to the characteristics of the main urban area of Chongqing. Therefore, this study adopted purpose-based sampling in the district and selected four primary schools in the district of Chongqing according to the differences in their geographical locations. A detailed description of this cohort study has been reported previously (24). The study was approved by the Medical Ethics Review Committee of Chongqing Medical University.

At baseline and the first follow-up, girls were examined for puberty outcomes, including breast and pubic hair development, while boys were examined for genital and pubic hair development by trained medical students. In addition, the Tanner stage was self-assessed by realistic color images (RCIs) and the PDS. The age, body weight, and height of the children were measured at baseline and the first follow-up. Father’s education, mother’s education, parental marital status, the number of left-behind children, and average monthly household income were surveyed at the first follow-up.

### 2.2 Physical examination

Pubertal development was assessed for boys and girls by trained medical school graduates during physical examinations according to Tanner stages (25). Before each investigation, every investigator received standard training from medical professors. In addition, each child's pubertal development was measured independently by two investigators for quality assurance. Tanner stages were determined as stage 1 (prepubertal), stage 2 (early pubertal), stage 3 (midpubertal), stage 4 (late pubertal), or stage 5 (postpubertal). PEs assessed pubertal development stages, including genital stages (G1–G5) for boys, breast stages (B1–B5) for girls, and pubic hair stages (P1–P5) for both genders (Supplementary Table S1).

### 2.3 Realistic color images

After completing the PDS and before undergoing the physical examination, puberty ratings were self-assessed using RCIs according to Tanner stages, as proposed by Carel and Leger in the *New England Journal of Medicine* in 2008 (26). Each child was presented with a gender-specific pubertal self-assessment questionnaire using realistic color images. After a short explanation of each stage of puberty by trained medical school graduates, the participants would read the questions along with the pictures and select the self-perceived stage that best represented their pubertal development by comparing their situation with the illustrations. Girls were asked to choose the most appropriate breast and pubic hair Tanner stage. Boys were asked to choose the most appropriate genital and pubic hair Tanner stage.

### 2.4 Pubertal Development Scale

Pubertal development was self-reported using the PDS before PE and RCIs. The PDS was developed to provide a continuous score of pubertal development and classified into a five-level category representing stages from prepubertal to postpubertal without physical examination or visually depicted sexual maturation stages (10). The PDS consists of five items: breast development and menstruation in girls; voice changes and facial hair growth in boys; and body hair growth, growth spurt, and skin changes in both sexes. Response options are as follows: not yet started (1), barely started (2), definitely started (3), and seems complete (4). In addition, girls’ menarche is a binary variable: no (0) or yes (1). The PDS scores can be used to classify individuals into five pubertal categories according to algorithms developed by Petersen et al. (1988) (10) and described by Carskadon and Acebo (27)—prepubertal, early pubertal, midpubertal, late pubertal, and postpubertal (in alignment with the original Tanner categories). The content of the PDS and the algorithm are provided in Table 1.

**Table 1:** Composition of the PDS and the computation of the PDS using the algorithm.

Questions: 1. Would you say that your growth in height: 2. And how about the growth of your body hair? (“Body hair” means hair any place other than your head, such as under your arms.) Would you say that your body hair growth: 3. Have you noticed any skin changes, especially pimples? Form for boys: 4. Have you noticed a deepening of your voice? 5. Have you begun to grow hair on your face? Form for girls: 4. Have you noticed your breasts have begun to grow? 5a. Have you begun to menstruate (started to have your period)? 5b. If yes, how old were you when you started to menstruate?		
Computation algorithm		
Tanner stages	Boys Totaling body hair growth, voice changes, and facial hair growth	Girls Totaling body hair growth, breast development, and menarche
1: Prepubertal	3	2 and no menarche
2: Early pubertal	4 or 5 (no 3-point responses)	3 and no menarche
3: Midpubertal	6, 7, or 8 (no 4-point responses)	3 and no menarche
4: Late pubertal	9–11	≤7 and menarche
5: Postpubertal	12	8 and menarche

### 2.5 Statistical analysis

The weighted kappa (wk) statistic was performed using R4.2.2, and other statistical analyses were performed using SPSS 25. McDonald's omega (28) coefficients were used to measure the internal consistency and scale reliability of the PDS. The wk statistic and accuracy were used to evaluate the agreement between PE and RCIs and between PE and the PDS. The strength of agreement criteria for wk is as follows: <0.00, poor; 0.00–0.20, slight; 0.21–0.40, fair; 0.41–0.60, moderate; 0.61–0.80, substantial; >0.8, almost perfect (29). Agreement between PE and the RCI or the PDS was calculated using Crosstabs, and percent (%) accuracy calculated the precise agreement between the three measurements of pubertal development. The Kendall rank correlation was also used to measure the strength of the correlations between the three measurements of pubertal development. The Kendall rank correlation coefficient ranges between −1 (perfect inverse relationship between two variables) and +1 (perfect direct relationship), with zero indicating a lack of relationship. The *t*-test and chi-squared test were used to analyze the influencing factors of PDS concordance.

## 3 Results

### 3.1 Characteristics of study participants

At baseline, a total of 1,234 participants, including 539 girls and 695 boys, were recruited for this study. At the first follow-up visit, 1,429 participants, including 734 girls and 695 boys, had completed the survey. Age, BMI, body weight, body height, father’s education, mother’s education, parental marital status, the number of left-behind children, and average monthly household income of the study participants are given in Table 2.

**Table 2:** Characteristics of study participants.

Variable	Frequency, *n* (%) or mean (SD)	
	Girls (*n*_0 _= 539, *n*_1 _= 734)	Boys = (*n*_0 _= 695, *n*_1 _= 695)
Age at baseline (years)	8.6 (1.2)	8.6 (1.2)
BMI^a^ at baseline		
Thinness	45 (8.4)	54 (7.8)
Normal	393 (73.0)	472 (68.2)
Overweight and obesity	100 (18.6)	166 (24.0)
Body weight at baseline (kg)	28.0 (6.8)	29.4 (7.6)
Body height at baseline (cm)	130.9 (9.3)	131.3 (8.8)
Age at the first follow-up (years)	9.3 (1.2)	9.2 (1.2)
BMI^a^ at the first follow-up		
Thinness	12 (1.6)	23 (3.3)
Normal	544 (74.2)	453 (65.2)
Overweight and obesity	177 (24.1)	219 (31.5)
Body weight at the 1st follow-up (kg)	31.6 (7.6)	32.7 (8.4)
Body height at the 1st follow-up (cm)	134.4 (9.1)	134.3 (8.6)
Father's education		
Middle school and below	337 (45.9)	311 (44.9)
High secondary school or above	397 (54.1)	382 (55.1)
Mother's education		
Middle school and below	360 (49.1)	334 (48.3)
High secondary school or above	373 (50.9)	357 (51.7)
Parental marital status		
Divorced	69 (9.4)	69 (9.9)
Married	662 (90.6)	625 (90.1)
Left-behind children^b^		
Yes	91 (12.4)	100 (14.4)
No	641 (87.6)	593 (85.6)
Average monthly household income (RMB)		
<2,000	226 (30.9)	213 (30.6)
2,001–4,000	329 (44.9)	320 (46.0)
>4,000	177 (24.2)	162 (23.3)

^a^ BMI (body mass index): standards published by the National Health and Family Planning Commission of the People's Republic of China in 2014 (document no. WS/T 456-2014, screening standard for malnutrition of school-age children and adolescents) and in 2018 (document no. WS/T 586-2018, screening for overweight and obesity among school-age children and adolescents).

^b^ Left-behind children: Rural registered minors under the age of 16 years whose parents are both migrant workers or one is a migrant worker and the other has no guardianship ability and who cannot normally live with their parents.

### 3.2 Comparison of PE and self-assessment by RCIs

**Figure 1:**
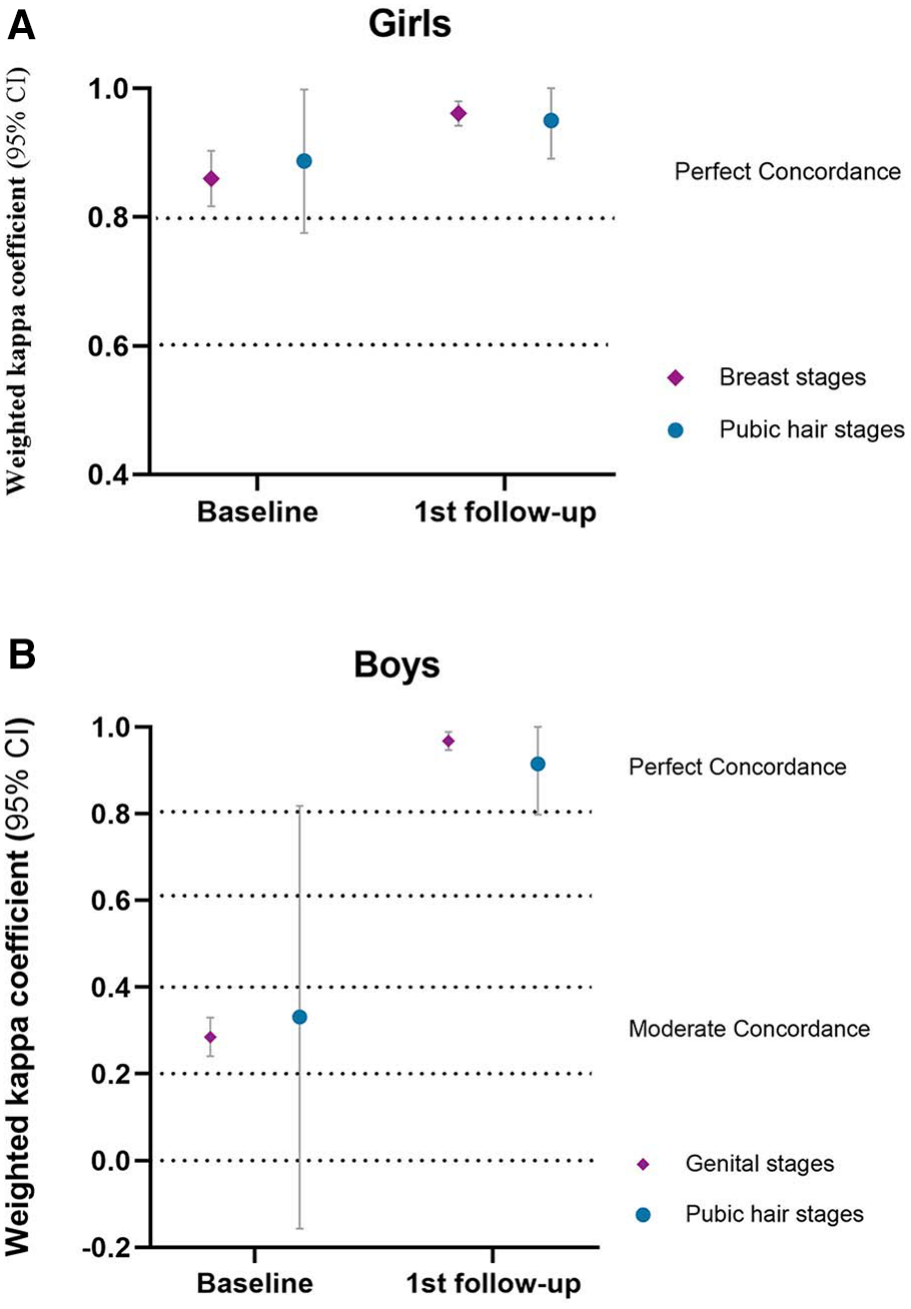
Concordance of PE and RCI.

Figure 1 shows the concordance of PE and self-assessment by RCIs for breast and pubic hair development in girls and genital and pubic hair development in boys.

For girls, there was almost perfect concordance between PE and RCIs. At baseline, the wk (weighted kappa coefficient) was 0.860 (*p *< 0.001) for breast development and 0.887 (*p *< 0.001) for pubic hair development. At the first follow-up visit, the wk was 0.961 (*p *< 0.001) for breast development and 0.950 (*p *< 0.001) for pubic hair development.

For boys, there was almost perfect concordance between PE and RCIs at the first follow-up visit and fair concordance in genital development at baseline. At baseline, the wk was 0.285 (*p *< 0.001) for genital development and 0.331 (95% CI −0.157 to 0.818) for pubic hair development. At the first follow-up visit, the wk was 0.968 (*p *< 0.001) for genital development and 0.915 (*p *< 0.001) for pubic hair development.

The accuracy, underestimation, overestimation, and Kendall rank correlation coefficient between PE and self-assessment by RCIs for breast/genital development and pubic hair development are presented in Supplementary Table S2.

### 3.3 Comparison of PE and self-reporting by the PDS

The McDonald's omega coefficient for all PDS items was 0.426 in girls and 0.105 for boys.

**Figure 2:**
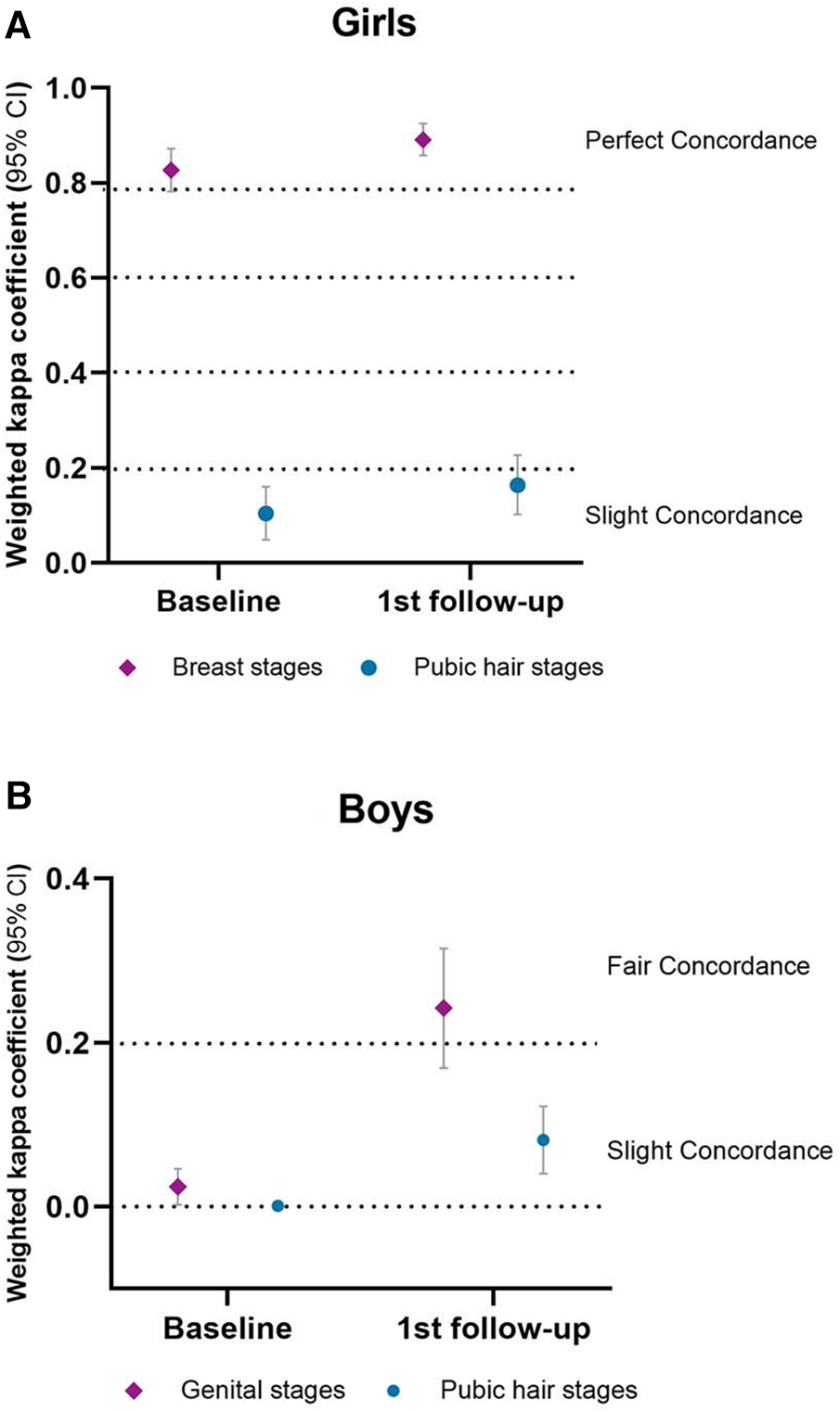
Concordance of PE and PDS.

For breast development in girls, the concordance between PE and self-reporting by the PDS was almost perfect at baseline (wk = 0.827, *p *< 0.001) and the first follow-up (wk = 0.891, *p *< 0.001). For pubic hair development in girls, there was slight concordance at baseline (wk = 0.104, *p *< 0.001) and the first follow-up (wk = 0.164, *p *< 0.001) (Figure 2).

For genital development comparison in boys, there was slight concordance (wk = 0.024, *p *< 0.05) at baseline and fair concordance (wk = 0.242, *p *< 0.001) at the first follow-up. For pubic hair development, there was poor concordance (wk = 0.001, *p > *0.05) at baseline and slight concordance (wk = 0.081, *p *< 0.001) at the first follow-up (Figure 2).

The accuracy, underestimation, overestimation, and Kendall rank correlation coefficient between PE and self-reporting by the PDS for breast/genital development and pubic hair development are presented in Supplementary Table S3.

### 3.4 Concordance between PE and PDS in age-stratified analysis

**Figure 3:**
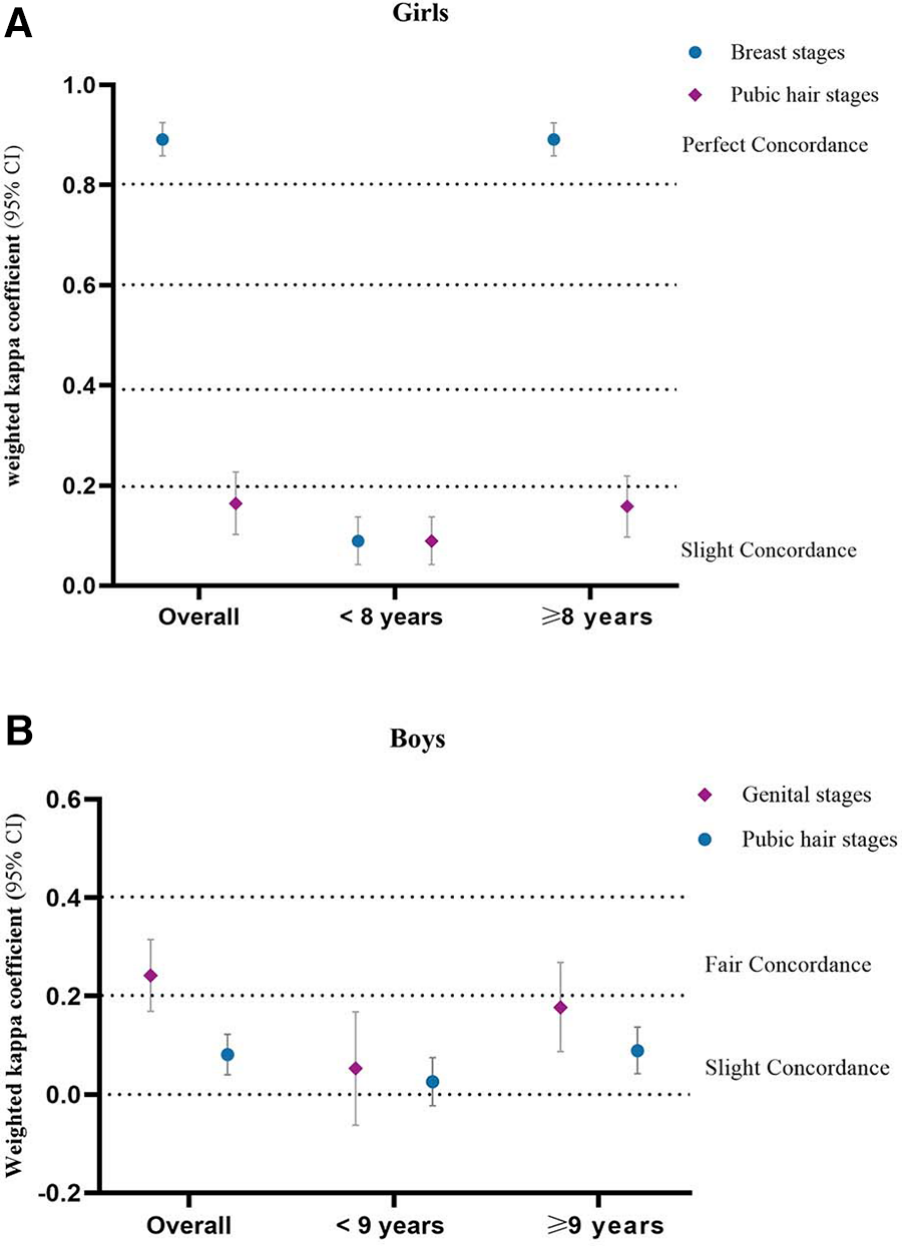
Concordance of PE and PDS in age-stratified analysis.

Age, BMI, father’s education, mother’s education, parental marital status, the number of left-behind children, and average monthly household income were included as influence factors in the analysis of the concordance between PE and the PDS. We found that age was the only factor affecting the poor concordance of the PDS in both genders (Supplementary Tables S4 and S5). The age of girls was categorized as <8 and ≥8 years old. The age of the boys was categorized as <9 and ≥9 years old (30). Ages classified as <8 years had slight concordance in girls for breast development, while ages classified as ≥8 years had perfect concordance. Ages classified as <8 and ≥8 years had slight concordance in girls for pubic hair development (Figure 3 and Supplementary Table S6). Ages classified as <9 and ≥9 years had slight concordance in boys for genital and pubic hair development (Figure 3 and Supplementary Table S7).

## 4 Discussion

To our knowledge, this is the first study to discuss the longitudinal reliability of pubertal development self-assessment using RCIs and the PDS and to compare the concordance of RCIs with the PDS through a longitudinal study. The current study has several key findings. First, our study suggests that self-assessment by RCIs for puberty development is a reliable and valid tool in longitudinal analysis. Second, the reliability and validity of the PDS were low, and the consistency of the PDS was not good. Third, the reliability of self-assessment by RCIs for puberty development is better than that by the PDS. Finally, the only factor affecting the poor concordance of the PDS in both sexes was age. These findings might assist doctors, healthcare providers, epidemiologists, and other researchers in deciding a more appropriate pubertal development self-assessment tool.

Our results demonstrate that RCIs are a reliable and valid pubertal development self-assessment tool. Like our results, studies from Thailand (31), Iran (32), and China (33, 34) have shown that such images can be used as a reliable tool to assess pubertal development in both sexes. Of these four studies, two included line drawings with brief explanatory text and two included colored images of Tanner stages; the colored images showed better concordance. Therefore, RCIs could be a better alternative pubertal assessment tool for extensive epidemiological research, (34) and might avoid the tension resulting from SMS examinations to improve the consent rate and reduce the dropout rate in cohorts.

Different from previous studies, we analyzed the dynamic changes in RCI self-rated reliability through longitudinal rather than cross-sectional data. Our longitudinal analysis suggested that it is more difficult for boys in early puberty to self-assess pubertal onset than girls and that boys tend to overestimate their genital development. Similarly, a Japanese study (35) showed that self-assessment of the onset of breast development using illustrations of Tanner stages in full color was consistent with assessment by PE in girls but not in boys. Moreover, a Danish study reported that boys overestimated their pubertal stage when using illustrations of pubertal stages (36). Yet, we noticed that the consistency of boys’ pubertal self-assessment using RCIs at the first follow-up visit was almost perfect. This might be because we explained puberty knowledge during our follow-up and boys became more knowledgeable about pubertal development, making the RCIs more reliable. We will continue to explore how sexual and reproductive health education enhances adolescents’ pubertal health and epidemiology studies into puberty. Therefore, for large-scale epidemiological studies requiring follow-up over many years, RCIs are a reliable alternative tool when SMS examinations are not feasible, saving manpower, budget, and time.

In addition to RCIs, we conducted the PDS reliability analysis using our cohort. Notably, our study suggests that the reliability and validity of the PDS are very low. Even so, the concordance of breast development self-assessment by the PDS was only almost perfect at baseline and the first follow-up visit, which might be because breast development is a representative indicator of pubertal development in girls (20). Studies from Bond et al. (15) and Pompeia et al. (14) reported that self-assessment of pubertal development using the PDS is useful when physical examination is not possible and images are unavailable. Also, the concordance of the PDS was between fair and moderate in the two studies. Such a conclusion could be challenged because the concordance of the PDS was not almost perfect or substantial. Another study (17) reported that the PDS was reliable and generally tracks with Tanner stages. However, it was only reliable when combining pubertal scores into three stages of development, and the original self-assessed Tanner stages by the PDS were fairly consistent. This means that the PDS is not applicable for measuring important indicators of pubertal development, such as pubertal onset and tempo, and cannot help doctors, healthcare providers, epidemiologists, and other researchers to accurately measure the pubertal development process. Hence, it is not known whether the concordance of the PDS is acceptable, and even if it is acceptable, the evaluation criteria are not uniform.

A study from Norris and Richter (18) did not recommend the PDS as a reliable self-assessment tool for pubertal development in South Africa, which is identical to our results. Another Chinese study (16) showed that Cronbach's alpha of the PDS was 0.80 for girls and 0.66 for boys in the Chinese version of the PDS. Although the study reported that the PDS was reliable, its reliability in boys was low. Also, there was only moderate concordance in self-rated pubertal development using the PDS. The translation and applicability of the PDS among Chinese children should be further investigated, combining our results of low reliability and validity and the influencing factors of PDS concordance. Currently, the PDS is widely used in large-scale epidemiological investigations (37–40). However, many studies have failed to find that the exact correspondence to Tanner stages between the PDS and PE is reliable (13, 17, 18). Therefore, the reliability of the PDS needs further study. Based on the results, our next step is to try out health education interventions and revise or develop a Pubertal Development Scale suitable for the local population.

## 5 Strengths and limitations

Our study had several strengths. First, we used a longitudinal study to assess the dynamic changes in the reliability of self-assessment tools for pubertal development. At present, most studies on the reliability of self-assessment tools for pubertal development are cross-sectional studies, which do not prove their applicability to long-term longitudinal studies. Second, we compared the agreement and reliability of self-assessment tools with the PDS. This comparison can help doctors, healthcare providers, epidemiologists, and other researchers decide a more appropriate pubertal development self-assessment tool. Third, current studies demonstrating good reliability of pubertal self-assessment tools predominantly involve white/Caucasian populations. There is limited research, including our study in China, on the reliability and validity of the PDS is relatively low, providing potential research directions in the region.

Several limitations should be acknowledged. First, although our study explored the reliability of pubertal self-assessment stools longitudinally, we conducted only two surveys that did not cover the whole of puberty, which limits the inferences of our results. Second, since each item can have an aggregate effect with the other items of the PDS and the Cronbach's alpha coefficient is very low, McDonald's omega was used to evaluate the internal consistency reliability of the PDS. However, the results showed that the reliability and validity of the PDS were very poor, which made the consistent results of the PDS in our study population unreliable. The possible reasons include the following: (1) Chinese children have relatively little understanding of pubertal development; (2) most of the children in this study are in the early stage of pubertal development (mainly stages 1, 2, and 3), with very few children in stages 4 and 5. In the next step of our research, we will conduct intervention studies on health education and revise or retranslate the Chinese version of the PDS.

## 6 Conclusions

The reliability of pubertal development self-assessment using RCIs is acceptable, while the reliability and validity of the PDS are low. This finding can assist doctors, healthcare providers, epidemiologists, and other researchers in deciding a more appropriate pubertal development self-assessment tool. Consequently, RCIs, rather than the PDS, are recommended as a reliable pubertal development self-assessment tool to measure pubertal development for large-scale epidemiological investigations and long-term longitudinal studies in China.

## Data Availability

The datasets presented in this article are not readily available because the data are stored and used among Chongqing Medical University research teams, and we are working on data sharing policies and a website for collaborators' data access. The datasets used or analyzed during the current study are available from the corresponding author on reasonable request. Requests to access the datasets should be directed to QL, liuqin@cqmu.edu.cn.
