# Supplementary material for: Validity of self-assessment pubertal Tanner stages by realistic color images and Pubertal Development Scale in a longitudinal cohort study
Source: Front Pediatr. 2024 Jul 16;12:1380934. doi: 10.3389/fped.2024.1380934 (PMC11286552; doi:10.3389/fped.2024.1380934)
Supplement: Supplementary file 1 [file Table1.docx]

**Supplementary Material**

S1 Table Tanner stages of breast development, genital development and pubic hair development

S2 Table Concordance and correlations of PE and RCI

S3 Table Concordance and correlations of PE and PDS

S4 Table Analysis of influence factors of PDS concordance in girls

S5 Table Analysis of influence factors of PDS concordance in boys

S6 Table PDS concordance of boys in age-stratified analysis

S7 Table PDS concordance of boys in age-stratified analysi

| **S1 Table** Tanner stages of breast development, genital development and pubic hair development | | |
| --- | --- | --- |
| Pubertal development | Tanner  stages | Standards |
| breast development for girls | B1 | Preadolescent: elevation of papilla only |
|  | B2 | Breast bud stage: elevation of breast and papilla as small mound. Areola diameter enlarged over stage 1 |
|  | B3 | Breast and areola both enlarged and elevated more than in stage 2, but with no separation of their contours |
|  | B4 | The areola and papilla form a secondary mound projecting above the contour of the breast |
|  | B5 | Mature stage: papilla only projects, with the areola recessed to the general contour of the breast |
| genital development for boys | G1 | Preadolescent. Testes, scrotum and penis are about same size and shape as in early childhood. |
|  | G2 | Scrotum slightly enlarged, with skin reddened and changed in texture. Little or no enlargement of penis at this stage. |
|  | G3 | Penis slightly enlarged, at first mainly in length. Scrotum further enlarged than in stage 2. |
|  | G4 | Penis further enlarged, with growth in breadth and development of glans. Scrotum further enlarged than in stage 3; scrotal skin darker than in earlier stages. |
|  | G5 | Genitalia adult in size and shape. |
| pubic hair development for both genders | P1 | Preadolescent. The vellus over the pubes is not further developed than that over the abdominal wall, i.e. no pubic hair. |
|  | P2 | Sparse growth of long, slightly pigmented downy hair, straight, or slightly curled, chiefly at the base of the penis or along the labia. |
|  | P3 | Considerably darker, coarser and more curled. The hair spreads sparsely over the junction of the pubes. |
|  | P4 | Hair now adult in type, but area covered is still considerably smaller than in the adult. No spread to the medial surface of thighs. |
|  | P5 | Adult in quantity and type with distribution of the horizontal (or classically 'feminine') pattern. Spread to medial surface of thighs but not up linea alba or elsewhere above the base of the inverse triangle (spread up linea alba occurs late and is rated stage 6). |

| **S2 Table** Concordance and correlations of PE and RCI | | | | | | | | | |
| --- | --- | --- | --- | --- | --- | --- | --- | --- | --- |
|  | PE | RCI | | | | | wk(95% CI) | Accuracy(Estimation Under/Over) | Kendall’s  τ-b |
|  |  | 1 | 2 | 3 | 4 | 5 |  |  |  |
| Baseline of girls | Breast stages |  |  |  |  |  | 0.860***  (0.817, 0.903) | 93.1%  (0.0% / 6.9%) | 0.886*** |
|  | 1 | **393** | 24 | 1 | 0 | 0 |  |  |  |
|  | 2 | 0 | **81** | 11 | 0 | 0 |  |  |  |
|  | 3 | 0 | 0 | **26** | 1 | 0 |  |  |  |
|  | 4 | 0 | 0 | 0 | **2** | 0 |  |  |  |
|  | 5 | 0 | 0 | 0 | 0 | **0** |  |  |  |
|  | Pubic hair stages |  |  |  |  |  | 0.887***  (0.775, 0.998) | 99.4%  (0.4% / 0.2%) | 0.955*** |
|  | 1 | **527** | 0 | 0 | 0 | 0 |  |  |  |
|  | 2 | 1 | **8** | 1 | 0 | 0 |  |  |  |
|  | 3 | 0 | 1 | **1** | 0 | 0 |  |  |  |
|  | 4 | 0 | 0 | 0 | **0** | 0 |  |  |  |
|  | 5 | 0 | 0 | 0 | 0 | **0** |  |  |  |
| 1st follow-up visit of girls | Breast stages |  |  |  |  |  | 0.961***  (0.942, 0.980) | 98.0%  (1.8% / 0.3%) | 0.971*** |
|  | 1 | **528** | 2 | 0 | 0 | 0 |  |  |  |
|  | 2 | 6 | **149** | 0 | 0 | 0 |  |  |  |
|  | 3 | 0 | 6 | **39** | 0 | 0 |  |  |  |
|  | 4 | 0 | 0 | 1 | **3** | 0 |  |  |  |
|  | 5 | 0 | 0 | 0 | 0 | **0** |  |  |  |
|  | Pubic hair stages |  |  |  |  |  | 0.950***  (0.891, 1.000) | 99.6%  (0.3% / 0.1%) | 0.930*** |
|  | 1 | **711** | 1 | 0 | 0 | 0 |  |  |  |
|  | 2 | 2 | **12** | 0 | 0 | 0 |  |  |  |
|  | 3 | 0 | 0 | **7** | 0 | 0 |  |  |  |
|  | 4 | 0 | 0 | 0 | **1** | 0 |  |  |  |
|  | 5 | 0 | 0 | 0 | 0 | **0** |  |  |  |
| Baseline of boys | Genital stages |  |  |  |  |  | 0.285***  (0.241, 0.330) | 57.3%  (0.1% / 42.6%) | 0.424*** |
|  | 1 | **235** | 281 | 4 | 0 | 0 |  |  |  |
|  | 2 | 1 | **160** | 10 | 0 | 0 |  |  |  |
|  | 3 | 0 | 0 | **2** | 0 | 0 |  |  |  |
|  | 4 | 0 | 0 | 0 | **0** | 0 |  |  |  |
|  | 5 | 0 | 0 | 0 | 0 | **0** |  |  |  |
|  | Pubic hair stages |  |  |  |  |  | 0.331  (-0.157, 0.818) | 99.4%  (0.4% / 0.1%) | 0.351*** |
|  | 1 | **688** | 1 | 0 | 0 | 0 |  |  |  |
|  | 2 | 3 | **1** | 0 | 0 | 0 |  |  |  |
|  | 3 | 0 | 0 | **0** | 0 | 0 |  |  |  |
|  | 4 | 0 | 0 | 0 | **0** | 0 |  |  |  |
|  | 5 | 0 | 0 | 0 | 0 | **0** |  |  |  |
| 1st follow-up visit of boys | Genital stages |  |  |  |  |  | 0.968***  (0.947, 0.989) | 98.7%  (1.3% / 0.0%) | 0.968*** |
|  | 1 | **502** | 0 | 0 | 0 | 0 |  |  |  |
|  | 2 | 9 | **181** | 0 | 0 | 0 |  |  |  |
|  | 3 | 0 | 0 | **3** | 0 | 0 |  |  |  |
|  | 4 | 0 | 0 | 0 | **0** | 0 |  |  |  |
|  | 5 | 0 | 0 | 0 | 0 | **0** |  |  |  |
|  | Pubic hair stages |  |  |  |  |  | 0.915***  (0.798, 1.000) | 99.7%  (0.3% /0.0%) | 0.919*** |
|  | 1 | **682** | 0 | 0 | 0 | 0 |  |  |  |
|  | 2 | 2 | **11** | 0 | 0 | 0 |  |  |  |
|  | 3 | 0 | 0 | **0** | 0 | 0 |  |  |  |
|  | 4 | 0 | 0 | 0 | **0** | 0 |  |  |  |
|  | 5 | 0 | 0 | 0 | 0 | **0** |  |  |  |
| **p<0.05,* ***p<0.01,* ****p<0.001, 95% CI = 95% Confidence interval, wk =* *weighted kappa coefficient, Kendall’s τ-b = Kendall rank correlation coefficient* | | | | | | | | | |

| **S3 Table** Concordance and correlations of PE and PDS | | | | | | | | | |
| --- | --- | --- | --- | --- | --- | --- | --- | --- | --- |
|  | PE | PDS | | | | | wk(95% CI) | Accuracy(Estimation Under/Over) | Kendall’s  τ-b |
|  |  | 1 | 2 | 3 | 4 | 5 |  |  |  |
| Baseline of girls | Breast stages |  |  |  |  |  | 0.827***  (0.782, 0.872) | 91.7%  (0.4% / 8.0%) | 0.882*** |
|  | 1 | **393** | 24 | 1 | 0 | 0 |  |  |  |
|  | 2 | 0 | **77** | 13 | 2 | 0 |  |  |  |
|  | 3 | 0 | 0 | **24** | 3 | 0 |  |  |  |
|  | 4 | 0 | 0 | 2 | **0** | 0 |  |  |  |
|  | 5 | 0 | 0 | 0 | 0 | **0** |  |  |  |
|  | Pubic hair stages |  |  |  |  |  | 0.104***  (0.0487, 0.160) | 72.9%  (0.0% / 27.1%) | 0.303*** |
|  | 1 | **393** | 101 | 31 | 2 | 0 |  |  |  |
|  | 2 | 0 | **0** | 9 | 1 | 0 |  |  |  |
|  | 3 | 0 | 0 | **0** | 2 | 0 |  |  |  |
|  | 4 | 0 | 0 | 0 | **0** | 0 |  |  |  |
|  | 5 | 0 | 0 | 0 | 0 | **0** |  |  |  |
| 1st follow-up visit of girls | Breast stages |  |  |  |  |  | 0.891***  (0.858,0.925) | 95.0%  (1.6% / 3.4%) | 0.947*** |
|  | 1 | **525** | 3 | 0 | 2 | 0 |  |  |  |
|  | 2 | 6 | **139** | 6 | 4 | 0 |  |  |  |
|  | 3 | 0 | 5 | **30** | 10 | 0 |  |  |  |
|  | 4 | 0 | 0 | 1 | **3** | 0 |  |  |  |
|  | 5 | 0 | 0 | 0 | 0 | **0** |  |  |  |
|  | Pubic hair stages |  |  |  |  |  | 0.164***  (0.102,0.227) | 72.6%  (0.0% / 27.4%) | 0.354*** |
|  | 1 | **531** | 147 | 26 | 8 | 0 |  |  |  |
|  | 2 | 0 | **0** | 10 | 4 | 0 |  |  |  |
|  | 3 | 0 | 0 | **1** | 6 | 0 |  |  |  |
|  | 4 | 0 | 0 | 0 | **1** | 0 |  |  |  |
|  | 5 | 0 | 0 | 0 | 0 | **0** |  |  |  |
| Baseline of boys | Genital stages |  |  |  |  |  | 0.024*  (0.002, 0.046) | 27.8%  (2.5% / 69.7%) | 0.054 |
|  | 1 | **76** | 331 | 110 | 0 | 0 |  |  |  |
|  | 2 | 15 | **116** | 40 | 0 | 0 |  |  |  |
|  | 3 | 0 | 2 | **0** | 0 | 0 |  |  |  |
|  | 4 | 0 | 0 | 0 | **0** | 0 |  |  |  |
|  | 5 | 0 | 0 | 0 | 0 | **0** |  |  |  |
|  | Pubic hair stages |  |  |  |  |  | 0.001  (0, 0.003) | 13.5%  (0.0% / 86.5%) | 0.053 |
|  | 1 | **91** | 447 | 148 | 0 | 0 |  |  |  |
|  | 2 | 0 | **2** | 2 | 0 | 0 |  |  |  |
|  | 3 | 0 | 0 | **0** | 0 | 0 |  |  |  |
|  | 4 | 0 | 0 | 0 | **0** | 0 |  |  |  |
|  | 5 | 0 | 0 | 0 | 0 | **0** |  |  |  |
| 1st follow-up visit of boys | Genital stages |  |  |  |  |  | 0.242***  (0.169, 0.315) | 67.7%  (14.0% / 16.6%) | 0.258*** |
|  | 1 | **393** | 100 | 9 | 0 | 0 |  |  |  |
|  | 2 | 102 | **75** | 13 | 0 | 0 |  |  |  |
|  | 3 | 0 | 1 | **2** | 0 | 0 |  |  |  |
|  | 4 | 0 | 0 | 0 | **0** | 0 |  |  |  |
|  | 5 | 0 | 0 | 0 | 0 | **0** |  |  |  |
|  | Pubic hair stages |  |  |  |  |  | 0.081***  (0.040，0.122) | 72.4%  (0.0% / 27.6%) | 0.235*** |
|  | 1 | **495** | 168 | 19 | 0 | 0 |  |  |  |
|  | 2 | 0 | **8** | 5 | 0 | 0 |  |  |  |
|  | 3 | 0 | 0 | **0** | 0 | 0 |  |  |  |
|  | 4 | 0 | 0 | 0 | **0** | 0 |  |  |  |
|  | 5 | 0 | 0 | 0 | 0 | **0** |  |  |  |
| **p<0.05,* ***p<0.01,* ****p<0.001, 95% CI = 95%*  *Confidence interval, wk =* *weighted kappa coefficient, Kendall’s τ-b = Kendall rank correlation coeffificient* | | | | | | | | | |

| **S4 Table** Analysis of influence factors of PDS concordance in girls | | | | | | | | |
| --- | --- | --- | --- | --- | --- | --- | --- | --- |
| Variable | Breast stages  Frequency, n (%) or mean (SD) | | | | Pubic hair stages  Frequency, n (%) or mean (SD) | | | |
|  | consistent | [inconsistent](javascript:;) | t/χ^2^ | *p* | consistent | [inconsistent](javascript:;) | t/χ^2^ | *p* |
| Age at 1st follow-up (years) | 8.6 (1.2) | 10.5 (0.8) | -7.231 | <0.001 | 8.8 (1.0) | 10.4 (0.8) | -19.689 | <0.001 |
| BMI at 1st follow-up |  |  |  |  |  |  |  |  |
| Thinness | 12 (100) | 0 | 3.045 | 0.218 | 11 (91.7) | 1 (8.3) | 7.563 | 0.023 |
| Normal | 520 (95.6) | 24 (4.4) |  |  | 405 (74.4) | 139 (25.6) |  |  |
| Overweight and Obesity | 164 (92.7) | 13 (7.3) |  |  | 116 (65.5) | 61 (34.5) |  |  |
| Father education |  |  |  |  |  |  |  |  |
| Middle school and below | 320 (95.0) | 17 (5.0) | 0.000 | 0.997 | 247 (73.3) | 90 (26.7) | 0.144 | 0.704 |
| High secondary school or above | 377 (95.0) | 30 5.0() |  |  | 286 (72.0) | 111 (28.0) |  |  |
| Mother education |  |  |  |  |  |  |  |  |
| Middle school and below | 343 (95.3) | 17 (4.7) | 0.156 | 0.692 | 258 (71.7) | 102 (28.3) | 0.296 | 0.587 |
| High secondary school or above | 353 (94.6) | 20 (5.4) |  |  | 274 (73.5) | 99 (26.5) |  |  |
| Parental marital status |  |  |  |  |  |  |  |  |
| Divorced | 65 (94.2) | 4 (5.8) | 0.089 | 0.765 | 49 (71.0) | 20 (29.0) | 0.094 | 0.760 |
| Married | 631 (95.0) | 33 (5.0) |  |  | 483 (72,7) | 181 (27.3) |  |  |
| Left-behind children |  |  |  |  |  |  |  |  |
| Yes | 88 (95.7) | 4 (4.3) | 0.106 | 0.745 | 71 (77.2) | 21 (22.8) | 1.099 | 0.294 |
| No | 609 (12.6) | 33 (10.8) |  |  | 462 (72.6) | 180 (27.4) |  |  |
| Average monthly household income (RMB) |  |  |  |  |  |  |  |  |
| ＜2000 | 215 (95.1) | 11 (4.9) | 0.172 | 0.917 | 165 (73.0) | 61 (27.0) | 1.937 | 0.380 |
| 2001-4000 | 313 (95.1) | 16 (4.9) |  |  | 232 (70.5) | 97 (29.5) |  |  |
| ＞4000 | 167 (94.4) | 10 (5.6) |  |  | 135 (76.3) | 42 (23.7) |  |  |

| **S5 Table** Analysis of influence factors of PDS concordance in boys | | | | | | | | |
| --- | --- | --- | --- | --- | --- | --- | --- | --- |
| Variable | Genital stages  Frequency, n (%) or mean (SD) | | | | Pubic hair stages  Frequency, n (%) or mean (SD) | | | |
|  | consistent | [inconsistent](javascript:;) | t/χ^2^ | *p* | consistent | [inconsistent](javascript:;) | t/χ^2^ | *p* |
| **Age at 1st follow-up (years)** | 9.0 (1.2) | 9.7 (1.1) | -7.554 | <0.001 | 9.0 (1.1) | 9.7 (1.2) | -6.703 | <0.001 |
| **BMI at 1st follow-up** |  |  |  |  |  |  |  |  |
| Thinness | 14 (60.9) | 9 (39.1) | 3.325 | 0.187 | 15 (65.2) | 8 (34.8) | 0.750 | 0.687 |
| Normal | 317 (70.0) | 136 (30.0) |  |  | 331 (73.1) | 122 (26.9) |  |  |
| Overweight and Obesity | 139 (63.5) | 80 (36.5) |  |  | 157 (71.7) | 62 (28.3) |  |  |
| **Father education** |  |  |  |  |  |  |  |  |
| Middle school and below | 211 (67.8) | 100 (32.2) | 0.025 | 0.874 | 226 (72.7) | 85 (27.3) | 0.002 | 0.964 |
| High secondary school or above | 257 (67.3) | 125 (32.7) |  |  | 277 (72.5) | 105 (27.5) |  |  |
| **Mother education** |  |  |  |  |  |  |  |  |
| Middle school and below | 229 (68.6) | 105 (31.4) | 0.206 | 0.650 | 248 (74.3) | 86 (25.7) | 0.991 | 0.320 |
| High secondary school or above | 239 (66.9) | 118 (33.1) |  |  | 253 (70.9) | 104 (29.1) |  |  |
| **Parental marital status** |  |  |  |  |  |  |  |  |
| Divorced | 53 (76.8) | 16 (23.2) | 2.981 | 0.084 | 49 (71.0) | 20 (29.0) | 0.082 | 0.774 |
| Married | 416 (66.6) | 209 (33.4) |  |  | 454 (72.6) | 171 (27.4) |  |  |
| **Left-behind children** |  |  |  |  |  |  |  |  |
| Yes | 68 (68.0) | 32 (32.0) | 0.007 | 0.931 | 74 (74.0) | 26 (26.0) | 0.154 | 0.694 |
| No | 402 (67.6) | 193 (85.6) |  |  | 429 (72.1) | 166 (27.9) |  |  |
| **Average monthly household income (RMB)** |  |  |  |  |  |  |  |  |
| ＜2000 | 144 (67.6) | 69 (32.4) | 1.757 | 0.415 | 154 (72.3) | 59 (27.7) | 1.512 | 0.469 |
| 2001-4000 | 210 (65.5) | 110 (34.4) |  |  | 226 (70.6) | 94 (29.4) |  |  |
| ＞4000 | 116 (71.6) | 46 (28.4) |  |  | 123 (75.9) | 39 (24.1) |  |  |

| **S6 Table** PDS concordance of boys in age-stratified analysis | | | | | | | |
| --- | --- | --- | --- | --- | --- | --- | --- |
|  | PE | PDS | | | | | wk(95% CI) |
|  |  | **1** | 2 | 3 | 4 | 5 |  |
| <8 years | Breast stages |  |  |  |  |  |  |
|  | 1 | **131** | 0 | 0 | 1 | 0 | 0.089 (0.042, 0.137) |
|  | 2 | 0 | **0** | 0 | 0 | 0 |  |
|  | 3 | 0 | 0 | **0** | 0 | 0 |  |
|  | 4 | 0 | 0 | 0 | **0** | 0 |  |
|  | 5 | 0 | 0 | 0 | 0 | **0** |  |
|  | Pubic hair stages |  |  |  |  |  |  |
|  | 1 | **131** | 0 | 0 | 1 | 0 | 0.089 (0.042, 0.137) |
|  | 2 | 0 | **0** | 0 | 0 | 0 |  |
|  | 3 | 0 | 0 | **0** | 0 | 0 |  |
|  | 4 | 0 | 0 | 0 | **0** | 0 |  |
|  | 5 | 0 | 0 | 0 | 0 | **0** |  |
| ≥8 years | Breast stages |  |  |  |  |  |  |
|  | 1 | **393** | 3 | 0 | 1 | 0 | 0.891 (0.858, 0.924) |
|  | 2 | 6 | **139** | 6 | 4 | 0 |  |
|  | 3 | 0 | 5 | **30** | 10 | 0 |  |
|  | 4 | 0 | 0 | 1 | **3** | 0 |  |
|  | 5 | 0 | 0 | 0 | 0 | **0** |  |
|  | Pubic hair stages |  |  |  |  |  |  |
|  | 1 | **399** | 147 | 26 | 7 | 0 | 0.158 (0.097, 0.219) |
|  | 2 | 0 | **0** | 10 | 4 | 0 |  |
|  | 3 | 0 | 0 | **1** | 6 | 0 |  |
|  | 4 | 0 | 0 | 0 | **1** | 0 |  |
|  | 5 | 0 | 0 | 0 | 0 | **0** |  |
| *wk = weighted kappa coefficient* | | | | | | | |

| **S7 Table** PDS concordance of boys in age-stratified analysis | | | | | | | |
| --- | --- | --- | --- | --- | --- | --- | --- |
|  | PE | PDS | | | | | wk(95% CI) |
|  |  | 1 | 2 | 3 | 4 | 5 |  |
| <9 years | Genital stages |  |  |  |  |  |  |
|  | 1 | **266** | 51 | 4 | 0 | 0 | 0.053 (-0.063, 0.168) |
|  | 2 | 9 | **2** | 0 | 0 | 0 |  |
|  | 3 | 0 | 0 | **1** | 0 | 0 |  |
|  | 4 | 0 | 0 | 0 | **0** | 0 |  |
|  | 5 | 0 | 0 | 0 | 0 | **0** |  |
|  | Pubic hair stages |  |  |  |  |  |  |
|  | 1 | **275** | 53 | 4 | 0 | 0 | 0.026 (-0.023, 0.075) |
|  | 2 | 0 | **0** | 1 | 0 | 0 |  |
|  | 3 | 0 | 0 | **0** | 0 | 0 |  |
|  | 4 | 0 | 0 | 0 | **0** | 0 |  |
|  | 5 | 0 | 0 | 0 | 0 | **0** |  |
| ≥9 years | Genital stages |  |  |  |  |  |  |
|  | 1 | **127** | 49 | 5 | 0 | 0 | 0.177 (0.087, 0.268) |
|  | 2 | 93 | **73** | 13 | 0 | 0 |  |
|  | 3 | 0 | 1 | **1** | 0 | 0 |  |
|  | 4 | 0 | 0 | 0 | **0** | 0 |  |
|  | 5 | 0 | 0 | 0 | 0 | **0** |  |
|  | Pubic hair stages |  |  |  |  |  |  |
|  | 1 | **220** | 115 | 15 | 0 | 0 | 0.089 (0.042, 0.137) |
|  | 2 | 0 | **8** | 4 | 0 | 0 |  |
|  | 3 | 0 | 0 | **0** | 0 | 0 |  |
|  | 4 | 0 | 0 | 0 | **0** | 0 |  |
|  | 5 | 0 | 0 | 0 | 0 | **0** |  |
| *wk = weighted kappa coefficient* | | | | | | | |
